# My sister's keeper?: genomic research and the identifiability of siblings

**DOI:** 10.1186/1755-8794-1-32

**Published:** 2008-07-25

**Authors:** Christopher A Cassa, Brian Schmidt, Isaac S Kohane, Kenneth D Mandl

**Affiliations:** 1 Children's Hospital Informatics Program at the Harvard-MIT Division of Health Sciences and Technology, Boston, MA, USA; 2 Clinical Decision Making Group, CSAIL, Massachusetts Institute of Technology, Cambridge, MA, USA; 3 Harvard Medical School, Boston, MA, USA

## Abstract

**Background:**

Genomic sequencing of SNPs is increasingly prevalent, though the amount of familial information these data contain has not been quantified.

**Methods:**

We provide a framework for measuring the risk to siblings of a patient's SNP genotype disclosure, and demonstrate that sibling SNP genotypes can be inferred with substantial accuracy.

**Results:**

Extending this inference technique, we determine that a very low number of matches at commonly varying SNPs is sufficient to confirm sib-ship, demonstrating that published sequence data can reliably be used to derive sibling identities. Using HapMap trio data, at SNPs where one child is homozygotic major, with a minor allele frequency ≤ 0.20, (N = 452684, 65.1%) we achieve 91.9% inference accuracy for sibling genotypes.

**Conclusion:**

These findings demonstrate that substantial discrimination and privacy risks arise from use of inferred familial genomic data.

## Background

Genomic data are increasingly integrated into clinical environments, stored in genealogical and medical records[1,2] and shared with the broader research community[3,4] without full appreciation of the extent to which these commodity level measurements may disclose the health risks or even identity of family members. While siblings, on average, share half of their contiguous chromosomal segments, well over half of a sibling's allelic values can be inferred using only population-specific allele frequency data and the genotypes of another sib. The informed consent process for research and clinical genomic data transmission must therefore include rigorous treatment of accurately quantified disclosure risks for all who will be impacted by such activity.

It is remarkably easy to positively identify a person with fewer than 40 independent, commonly varying SNPs, using a physical sample or a copy of those values[5]. As DNA sequences cannot be revoked or changed once they are released, any disclosure of such data poses a life-long privacy risk. Unlike conventional fingerprints, which provide little direct information about patients or relatives, SNP genotypes may encode phenotypic characteristics, which can link sequences to people[6]. Despite these privacy issues[7,8], use of genetic sequencing is increasing in both forensics[9] and clinical medicine. The recent genetic fingerprinting provision in the renewal of the federal Violence Against Women Act[10], alone, may result in one million new sequenced individuals each year, markedly increasing the number of available links between identities and genotypes. This genetic fingerprinting has an impact on people beyond those directly sequenced–genetic testing partially reveals genotypes of siblings and other family members.

At each locus in a child's genome, each parent transmits only one of his or her two chromosomes. If we have the genotype of one child, and would like to use that information to help infer the genotype of a sibling, we consider both the known parental genotypes (for the alleles they have transmitted to their first sibling,) and also consider those chromosomes they have but have not transmitted. We assume that the unknown parental alleles are drawn from a reference population, such as one of the HapMap populations. Now, considering the genotype of the inferred sibling (2^nd ^child), with probability 0.25, the sibling will receive the same 2 chromosomes transmitted to the first child, in which case they will have the same genotype. With probability 0.25, the inferred sibling will receive both previously untransmitted chromosomes, in which case the sibling will have the same genotype distribution as the reference population. If only one of the same chromosomes is transmitted, then one chromosome will be the same and the other will be drawn from the population.

## Methods

To quantify the risk of SNP disclosure to relatives, we demonstrate a model for inferring sibling genotypes using proband SNP data and population-specific allele frequency databases, such as the HapMap[10,11]. We also evaluate the probability that two people, in a selected pool of individuals, are siblings given a match at an independent subset of SNPs, and show that this number can be made remarkably low with appropriate SNP selection.

### Enhanced ability to infer sibling genotypes

First, consider the case where one sibling's genotype is known to be *'AA'*, and the goal is to determine the probability that a second sibling's genotype will also be *'AA' *at that locus. Because there is additional knowledge–the familial relationship between the two sibs–the prior probability of the second sib carrying a specific genotype at a selected SNP will be altered under the new constraint. A conditional probability expression that sums over the nine possible parental genotypic combinations (for example, maternal genotype *'Aa' *with paternal genotype *'AA'*) at a single SNP, each denoted as *i *can be used:

$$\begin{array}{l} p(Sib_{2}AA|Sib_{1}AA)=\sum_{i=1}^{9}p(Sib_{2}AA|parental\;comb.\;i)p(parental\;comb.\;i|Sib_{1}AA)\\ =\sum_{i=1}^{9}\frac{p(Sib_{2}AA\cap parental\;comb.\;i)}{p(parental\;comb.\;i)}p(parental\;comb.\;i|Sib_{1}AA) \end{array}$$

where *Sib*_1_*AA *and *Sib*_2_*AA *refer to Sib_1 _and Sib_2 _genotypes *'AA' *at a selected SNP, respectively.

**Figure 1:**
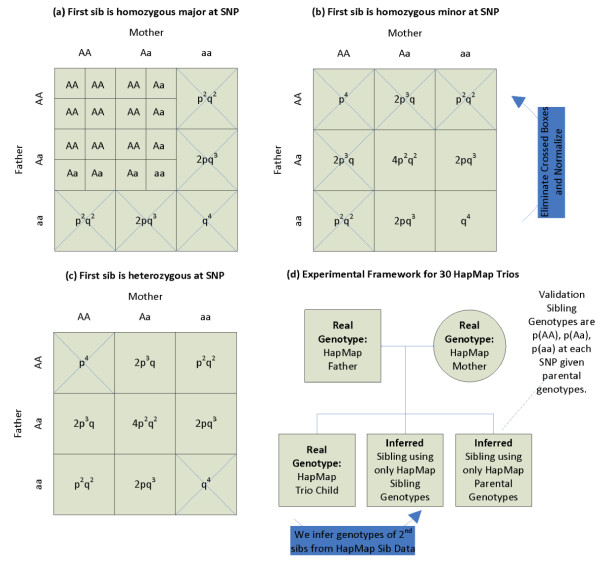
(a-c) Refining mechanism for homozygous major SNPs: when the first sibling is homozygous major (a), homozygous minor (b), or heterozygous (c) at a given SNP, this constrains the possible parental genotypes; in the first case, five of nine parental genotypic combinations can be eliminated (crossed boxes). Using HapMap CEPH SNP population frequencies, *p *and *q*, the probability frequencies are populated for the remaining squares, and normalized. The probability that subsequent sibs will be homozygous major, heterzygous, or homozygous minor can then be calculated using the probabilities that parents would contribute specific allelic values. (d) For each of 30 HapMap CEPH trios, the Sib_1 _genotype and the SNP population frequencies are used (without the parent genotypes) to infer *p('AA'), p('Aa')*, and *p('aa') *for subsequent siblings. Those probabilities are then validated against those that would be expected given only the parental genotypes at each SNP.

With unknown parental genotypes, we would calculate *p(Sib*_2_*AA) *considering all nine possible parental genotype combinations, but knowledge that Sib_1 _has genotype *'AA' *allows exclusion of any parental combinations where either parent has genotype *'aa'*, as that would require the transmission of at least one copy of the *'a' *allele to Sib_1_, if non-paternity and new mutations are excluded. HapMap SNP population frequencies, *p *and *q*, for each selected SNP, can be used to calculate the probabilities of each parental combination, *i*. Once these values have been calculated, the genotype of the first sibling eliminates possible parental genotypic candidates (Figs. 1A–C), and the remaining probabilities are normalized.

### Measuring the information content of Sibling genotype data

When calculating the probability of a specific Sib_2 _genotype given a known Sib_1 _genotype, it is possible to directly measure the benefit of the proband genotype information in improving Sib_2 _inferences. This involves measuring the difference between the prior Hardy-Weinberg probability for the genotype, given only population frequencies, and the posterior probability, as calculated by the conditional expression above. To measure the information content provided by the first sibling's genotype, we propose the use of a likelihood ratio test statistic, comparing models where two individuals are known to be siblings versus two individuals that are known to be unrelated. There are a total of nine possible likelihood ratios, Λ_Ind1, Ind2 genotypes_, for each of the possible individual genotypic combinations, such as *Ind*_1 _*AA*:

$$\begin{array}{l} \Lambda_{Ind_{1},Ind_{2}\;genotypes}=\frac{p(Ind_{2}genotype|Ind_{1}genotype\cap siblings)}{p(Ind_{2}genotype|Ind_{1}genotype\cap unrelated)}\\ =\frac{p(Sib_{2}genotype|Sib_{1}genotype\cap siblings)}{\left(\frac{p(Ind_{2}genotype\cap p(Ind_{1}genotype\cap unrelated)}{p(Ind_{1}genotype\cap unrelated)}\right)}\\ =\frac{\sum_{i=1}^{9}p(Sib_{2}genotype|parental\;comb.\;i)p(parental\;comb.\;i|Sib_{1}genotype)}{\left(\frac{p(Ind_{2}genotype\cap p(Ind_{1}genotype\cap unrelated)}{p(Ind_{1}genotype\cap unrelated)}\right)}\\ =\frac{\sum_{i=1}^{9}\frac{p(Sib_{2}genotype\cap parental\;comb.\;i)}{p(parental\;comb.\;i)}p(parental\;comb.\;i|Sib_{1}genotype)}{\left(\frac{p(Ind_{2}genotype)\cdot p(Ind_{1}genotype)\cdot (1-\frac{1}{N})}{p(Ind_{1}genotype)\cdot (1-\frac{1}{N})}\right)}\\ ≅\frac{\sum_{i=1}^{9}\frac{p(Sib_{2}genotype\cap parental\;comb.\;i)}{p(parental\;comb.\;i)}p(parental\;comb.\;i|Sib_{1}genotype)}{p(Ind_{2}genotype)} \end{array}$$

**Figure 2:**
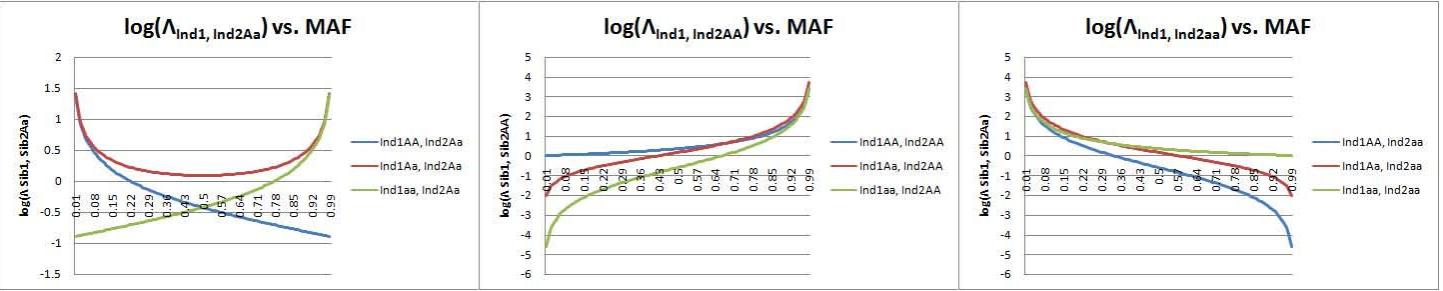
Log likelihood ratio test statistic for sibling inferences: for each Sib_1 _genotype, the log likelihood ratio for each possible Sib_2 _inference is shown versus MAF. These charts describe how informative the Sib_1 _genotype is when inferring each Sib_2 _genotype.

The denominator becomes *p(Ind*_2 _*genotype*), which is either *p*^2^, *2pq*, or *q*^2^. This is intuitive; when considering two unrelated individuals, the probability that the 2^nd ^has a specific genotype can only be identified using the population frequencies for that genotype. The numerator is the posterior probability expression derived in Table 1, also in terms of *p *and *q*. The log of this odds ratio can then be used as a statistic for measuring relatedness, depending only on the SNP allele frequency and the Sib_1 _genotype (Fig. 2).

**Table 1:** Sib_2 _inference error reduction when Sib_1 _genotype is known.

**Sib**_2_	**Sib**_1_	**Prior Prob.**	**Posterior Prob.**	**Error Reduction**
**AA**	AA	p^2^	p^2 ^+ pq + 1/4q^2^	|p^2 ^- [p^2 ^+ pq + 1/4q^2^]|
**Aa**	AA	2pq	pq + 1/2q^2^	|2pq - [pq + 1/2q^2^]|
**aa**	AA	q^2^	1/4q^2^	|q^2 ^- [1/4q^2^]|
**AA**	Aa	p^2^	1/2p^2 ^+ 1/4pq	|p^2 ^- [1/2p^2 ^+ 1/4pq]|
**Aa**	Aa	2pq	1/2p^2 ^+ (2/3)^-1^pq + 1/2q^2^	|2pq - [1/2p^2 ^+ (2/3)^-1^pq + 1/2q^2^]|
**aa**	Aa	q^2^	1/4pq + 1/2q^2^	|q^2 ^- [1/4pq + 1/2q^2^]|
**AA**	Aa	p^2^	1/4p^2^	|p^2 ^- [1/4p^2^]|
**Aa**	Aa	2pq	1/2p^2^+pq	|2pq - [1/2p^2^+pq]|
**aa**	Aa	q^2^	1/4p^2 ^+ pq + q^2^	|q^2 ^- [1/4p^2 ^+ pq + q^2^]|

The error reduction depends only on the allele frequencies, and at all frequencies, the error is reduced, improving the quality of genotypic inference.

The allele frequency, *p*, that maximizes this statistic can then be found numerically for each Λ_Ind1, Ind2 genotypes _expression, to identify which allele frequencies and conditions are most informative for genotypic inferences. These results are below in Table 2.

**Table 2:** Finding the MAF that maximizes the log likelihood ratio test statistic for each Sib_2 _genotypic inference type.

**Sib**_2_	**Sib**_1_	**Maximizing MAF**	**Log(Λ**_Ind1, Ind2 genotypes_)
AA	AA	0.01	3.407
Aa	AA	0.01	3.699
aa	AA	0.01	3.389
AA	Aa	0.99	1.396
Aa	Aa	0.01, 0.99	1.407
aa	Aa	0.01	1.396
AA	aa	0.99	3.389
Aa	aa	0.99	3.699
aa	aa	0.99	3.407

The *maximizing MAF *is the allele population frequency at which the most information will be derived about the Sib_2 _genotype from Sib_1 _under that Sib genotypic combination. Note: There are two equally maximizing MAF values for Log(Λ_Sib1Aa, Sib2Aa_), 0.01 and 0.99, both resulting in a value of 1.407.

### Confirming sib-ship with two non-matching sets of SNP genotypes

The above inference technique can be extended to confirm sib-ship in two non-matching samples of SNP sequence data. Given a set of matches at *M *independent loci from a pool of *N *individuals, an expanded form of Bayes Theorem can be used to calculate *p(sibs|match at M loci) *directly:

$$\begin{array}{l} p(sibs|match\;at\;M\;loci)=\frac{p(match\;at\;M\;loci|sibs)p(sibs)}{p(match\;at\;M\;loci|sibs)p(sibs)+p(match\;at\;M\;loci|!sibs)p(!sibs)}\\ =\frac{{\left[p(both\;AA|sibs)+p(both\;Aa|sibs)+p(both\;aa|sibs)\right]}^{M}\left(\frac{1}{N}\right)}{{\left[p(both\;AA|sibs)+p(both\;Aa|sibs)+p(both\;aa|sibs)\right]}^{M}\left(\frac{1}{N}\right)+p{\left(match|!sibs\right)}^{M}\left(1-\frac{1}{N}\right)} \end{array}$$

**Figure 3:**
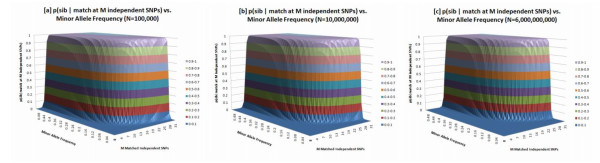
Sib-ship identifiability surfaces: these surfaces describe the probability of sib-ship as a function of *M*, the number of partially matched independent SNPs (between two individuals) and MAF. We show this across three sample size pools–N = (a)100,000; (b)10,000,000; (c)6,000,000,000 people. At high MAFs even very large increases in the potential sample pool size will not prevent sib-ship confirmation with relatively few matched SNPs. For example, if loci with MAF = 0.25 are selected, the number of matched SNPs to confirm sib-ship with p = 0.999 is 50 with a candidate pool of 100,000 and increases to only 80, in a group of 6 billion.

*p(match|!sibs) *can be calculated for each SNP using the population frequency; it is the probability that two unrelated individuals in the population would share the same genotype, *'AA'*, *'Aa'*, or *'aa'*. The expression *p(match|!sibs) *is effectively the same as *p(match) *as long as the sample pool, *N*, is large enough, as the probability of sib-ship is very low in a large pool. For three different pool sizes, (*N = 100,000;10,000,000;6,000,000,000*), we have created a sib-ship probability surface that varies with the number of matched SNPs and MAF of those SNPs (Fig. 3) and published supporting values for these probabilities in Table 3. For SNPs that commonly vary in the population, a small number of genotypic matches are required to confirm sib-ship.

**Table 3:** Probability of sib-ship for three pool sizes.

***N *= 100,000**										
**q**	**M = 1**	**M = 10**	**M = 20**	**M = 30**	**M = 40**	**M = 50**	**M = 60**	**M = 70**	**M = 80**	**M = 90**

**0**	0.00001	1.00E-05	1.00E-05	1.00E-05	1.00E-05	1.00E-05	1.00E-05	1.00E-05	1.00E-05	1.00E-05
**0.05**	1.10E-05	2.67E-05	7.11E-05	0.000189	0.000505	0.001345	0.003578	0.009482	0.024886	0.063706
**0.1**	1.21E-05	6.64E-05	0.000441	0.002923	0.019099	0.114527	0.462126	0.850907	0.974301	0.996045
**0.15**	1.31E-05	0.000148	0.002194	0.031572	0.325877	0.87757	0.990679	0.999366	0.999957	0.999997
**0.2**	1.40E-05	0.000287	0.008152	0.190701	0.871059	0.994863	0.99982	0.999994	1	1
**0.25**	1.47E-05	0.000472	0.021816	0.512966	0.980292	0.999574	0.999991	1	1	1
**0.3**	1.52E-05	0.000666	0.042483	0.747176	0.994946	0.999924	0.999999	1	1	1
**0.35**	1.55E-05	0.000823	0.063574	0.848341	0.997835	0.999974	1	1	1	1
**0.4**	1.57E-05	0.000924	0.078846	0.88788	0.998637	0.999985	1	1	1	1
**0.45**	1.58E-05	0.000975	0.086919	0.902796	0.998898	0.999989	1	1	1	1
**0.5**	1.58E-05	0.000989	0.089295	0.906621	0.998961	0.999989	1	1	1	1

**N = 10,000,000**										

**q**	**M = 1**	**M = 10**	**M = 20**	**M = 30**	**M = 40**	**M = 50**	**M = 60**	**M = 70**	**M = 80**	**M = 90**

**0**	1.00E-07	1.00E-07	1.00E-07	1.00E-07	1.00E-07	1.00E-07	1.00E-07	1.00E-07	1.00E-07	1.00E-07
**0.05**	1.10E-07	2.67E-07	7.11E-07	1.89E-06	5.05E-06	1.35E-05	3.59E-05	9.57E-05	0.000255	0.00068
**0.1**	1.21E-07	6.64E-07	4.41E-06	2.93E-05	0.000195	0.001292	0.008518	0.053991	0.274896	0.715775
**0.15**	1.31E-07	1.48E-06	2.20E-05	0.000326	0.004811	0.066884	0.515231	0.940333	0.995739	0.999711
**0.2**	1.40E-07	2.87E-06	8.22E-05	0.002351	0.063279	0.659483	0.982308	0.999372	0.999978	0.999999
**0.25**	1.47E-07	4.72E-06	0.000223	0.010423	0.332172	0.959166	0.999099	0.999981	1	1
**0.3**	1.52E-07	6.66E-06	0.000443	0.028705	0.663129	0.992431	0.999886	0.999998	1	1
**0.35**	1.55E-07	8.24E-06	0.000678	0.052974	0.821712	0.997374	0.999968	1	1	1
**0.4**	1.57E-07	9.25E-06	0.000855	0.073378	0.879899	0.998527	0.999984	1	1	1
**0.45**	1.58E-07	9.76E-06	0.000951	0.084983	0.900612	0.99887	0.999988	1	1	1
**0.5**	1.58E-07	9.90E-06	0.00098	0.088497	0.905783	0.998951	0.999989	1	1	1
**N = 6,000,000,000**										

**q**	**M = 1**	**M = 10**	**M = 20**	**M = 30**	**M = 40**	**M = 50**	**M = 60**	**M = 70**	**M = 80**	**M = 90**

**0**	1.60E-10	1.67E-10	1.67E-10	1.67E-10	1.67E-10	1.67E-10	1.67E-10	1.67E-10	1.67E-10	1.67E-10
**0.05**	1.80E-10	4.44E-10	1.18E-09	3.16E-09	8.42E-09	2.24E-08	5.98E-08	1.60E-07	4.25E-07	1.13E-06
**0.1**	2.00E-10	1.11E-09	7.35E-09	4.89E-08	3.25E-07	2.16E-06	1.43E-05	9.51E-05	0.000631	0.00418
**0.15**	2.10E-10	2.47E-09	3.66E-08	5.43E-07	8.06E-06	0.000119	0.001768	0.025594	0.280299	0.852397
**0.2**	2.30E-10	4.78E-09	1.37E-07	3.93E-06	0.000113	0.003217	0.084701	0.726254	0.987023	0.999542
**0.25**	2.40E-10	7.87E-09	3.72E-07	1.76E-05	0.000828	0.037674	0.648979	0.988676	0.999758	0.999995
**0.3**	2.50E-10	1.11E-08	7.39E-07	4.93E-05	0.00327	0.179341	0.935717	0.99897	0.999985	1
**0.35**	2.50E-10	1.37E-08	1.13E-06	9.32E-05	0.007623	0.387598	0.981185	0.999767	0.999997	1
**0.4**	2.60E-10	1.54E-08	1.43E-06	0.000132	0.012063	0.530447	0.990523	0.999897	0.999999	1
**0.45**	2.60E-10	1.63E-08	1.59E-06	0.000155	0.014878	0.595717	0.993092	0.999929	0.999999	1
**0.5**	2.60E-10	1.65E-08	1.63E-06	0.000162	0.01577	0.613392	0.993675	0.999936	0.999999	1

In a sample pool of size *N*, provided below, the probability that two individuals are siblings given a match at a subset of SNPs is charted as a function of *M*, the number of independent SNPs that they match at, and the minor allele frequency, *q*.

### Modeling a series of SNP inferences using a binomial distribution

A binomial distribution can be used to represent a series of sibling genotypic inferences, such as the probability of correct inferences at 50 SNP loci, if each inference meets specific criteria. Independent inferences can be treated as a random variable with probability *p *of success, as long as independent SNPs are selected, with the same MAF and Sib_1 _genotype.

$$p(k,n,p)=\left(\begin{array}{c} n\\ k \end{array}\right)p^{k}{\left(1-p\right)}^{n-k}$$

where *p(k, n, p) *refers to the probability that *k *correct inferences were made out of *n *attempted inferences when the probability of success for each inference attempt is *p*. This measure will enable those who attempt to infer SNP genotypes to calculate the probability of matching at a subset of independent SNPs.

The cumulative binomial measures the probability of reaching up to *k *successes in *n *trials with probability *p *of success at each attempt:

$$F(k;n,p)=P(X\leq k)=\sum_{j=0}^{k}\left(\begin{array}{c} n\\ j \end{array}\right)p^{j}{\left(1-p\right)}^{n-j}$$

If *n *guesses are considered (i.e. *n *SNPs are genotyped and used for sib inference), *F(k, n, p) *is the probability that at least *k *of those will be correct.

## Results

### Validation of SNP genotype inference using HapMap trio data

We then empirically infer sibling genotypic sequences from HapMap trio child genotypes using the above technique. At 700,000 SNP loci on chromosomes 2, 4, and 7, in each of 30 HapMap CEPH trios, the trio sibling, Sib_1_, known genotypes are combined with the CEPH and global HapMap SNP allele frequencies to produce genotypic inferences of a hypothetical sib, Sib_2_, at these loci. The inference method produces three genotypic probabilities for Sib_2 _(or subsequent siblings): *p(Sib*_2_*AA|Sib*_1_*genotype)*, *p(Sib*_2_*Aa|Sib*_1_*genotype)*, and *p(Sib*_2_*aa|Sib*_1_*genotype) *for each SNP, which we call the SNP probability vector.

**Figure 4:**
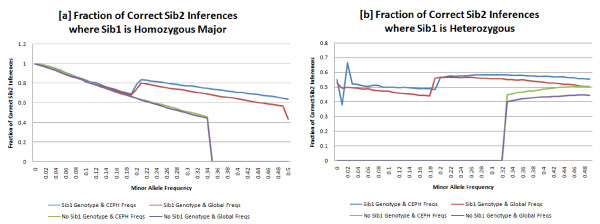
Fraction of correct Sib2 inferences: the fraction of Sib2 SNPs that can be correctly identified when Sib1 is (a)homozygous major or (b)heterozygous. Each line represents use of distinct data–inclusion or exclusion of Sib1 genotypes, and use of population-specific or global allele frequency data. Without Sib1 genotypes, homozygous major inferences would always be incorrect at MAF ≥ 0.33 and heterozygous inferences would always incorrect at MAF ≤ 0.33. At many allele frequencies, use of Sib1 genotypes dramatically improves Sib2 inferences.

The ability to correctly infer a sibling genotype from a trio child genotype can be validated by comparing whether the best estimated genotype, using only the sibling genotype and population frequencies, matches the best estimated genotype using the parental genotypic data (Fig. 1D). We do this by comparing the plural, largest, value in the SNP probability vector, with the plural value in the SNP probability vector that would be expected given the parental genotypes and Mendelian Inheritance. The fraction of correct inferences for SNPs where the Sib_1 _is homozygous major or heterozygous versus MAF are graphed in Figs. 4A–B, respectively. There were insufficient SNPs where the trio child was homozygous minor, so they have been excluded from this analysis. The appendix contains details about the HapMap population used as well as the distance and scoring metric used.

For inferences at SNPs where the trio child, Sib_1_, was homozygous major, with MAF < 0.05 (N = 300512,43.2%), we are able to correctly infer the genotype of other siblings, e.g. Sib_2_, with 98.5% accuracy when using population-specific allele frequency data. At SNPs with MAF < 0.20 (N = 452684,65.1%) we achieve 91.9% average accuracy. For SNPs where the first sibling is heterozygous, with MAF > 0.20 (N = 125796,18.1%), it is possible to infer the correct genotype of the second sibling with 57.7% average accuracy. Without Sib_1 _genotypes, all inferences for homozygous major SNPs with MAF ≥ 0.33 and heterozygous SNPs with MAF ≤ 0.33 would be incorrect when validated against plural parental values. At these allele frequencies, as well as others, use of Sib_1 _genotypes markedly improves Sib_2 _inferences.

### Deriving propensity to disease from sibling SNP data

Additionally, sibling SNP data can be used to quantify an individual's disease propensity through genotypic inference, without that individual's actual sequence data. For example, the likelihood ratio test statistic above may also be used to describe relative risk, using a multiplicative model.

$$\begin{array}{l} \Gamma_{Sib_{2}genotype|Sib_{1}genotype}=\frac{probability\;with\;sibling\;knowledge}{probability\;without\;sibling\;knowledge}\\ =\frac{p(Sib_{2}genotype|Sib_{1}genotype)}{p(Sib_{2}genotype)}\\ =\frac{\sum_{i=1}^{9}\frac{p(Sib_{2}genotype\cap parental\;comb.\;i)}{p(parental\;comb.\;i)}p(parental\;comb.\;i|Sib_{1}genotype)}{p(Sib_{2}genotype)} \end{array}$$

For example, the relative risk of *Sib*_2_*Aa*, carrying one copy of the disease allele 'a', is provided by information from the Sib_1_aa genotype:

ΓAa|Sib1aa=p(Sib2Aa|Sib1aa)p(Sib2Aa)=12p2+pq2pq=12p+(1−p)2(1−p)=1−12p2−2p

In this example, at MAF = 0.01, the relative risk of genotype 'Aa' is 25.25, given information that Sib_1 _carries genotype 'aa' at that locus. However, at MAF = 0.5, the relative risk of genotype 'Aa' is 0.75, given information that Sib_1 _carries genotype 'aa', explaining that the risk of having the genotype 'Aa' is reduced at this MAF. This may seem counterintuitive, as the risk of carrying a disease allele is actually higher at this MAF, but Sib_2 _carrying genotype 'Aa' is lower than in the control population, while the relative risk of carrying the disease allele with genotype 'aa' is higher.

Γaa|Sib1aa=p(Sib2aa|Sib1aa)p(Sib2aa)=14p2+pq+q2q2=14p2+p(1−p)+(1−p)2(1−p)2

At MAF 0.5, Γ_aa|Sib1aa _is 2.25, demonstrating that it is more likely that a disease allele will be carried by Sib_2 _in genotype 'aa' than in the control population given the Sib_1 _genotype.

The explicit probability of developing a disease is also altered. If an individual with genotype *'Aa' *at a specific locus has a probability *p*_*d *_of developing a disease by age *a*, and that individual has a probability *p*_*s *_of having that genotype given his sibling's genotype at that locus, his probability of developing that disease by age *a *is *p*_*s*· _*p*_*d*_. This can easily be extended to multiple independent loci, important for diseases in which a set of common or rare variants dictates disease likelihood[12,13]. As SNPs are both clinically informative and there is a wealth of supporting allele frequency data, they have been the focus of our analysis, however there are other genomic data types which should be considered in a rigorous privacy and propensity analysis, including copy number variant and mutation data.

## Discussion

These findings demonstrate that substantial discrimination and privacy concerns arise from use of inferred familial genomic data. While the Genetic Information Nondiscrimination Act of 2008 (GINA, H.R. 493), recently passed into law, would mitigate the threat of direct discriminatory action by employers or insurers[14], there will continue to be other uses of genomic data that pose privacy risks, including the use of genetic testing in setting life, disability, and long-term care insurance premiums[15]. Familial genotypic sequences can be used to assist in forensic or criminal investigations for indirect identification of genotype, increasing the number of people who may be identified[16,17]. Similarly, Freedom of Information Act (FOIA)[18] requests related to federally-funded genome wide association studies could potentially be used to identify research participants and their family members. Clinically, choosing the detail and type of disease propensity information that must be disclosed to patients and their potentially affected family members is also under debate[19,20].

Quantifying the information content of disclosed genomic data will add clarity to the informed consent process when a patient shares genotypic data for research use. For research investigations, it is conceivable that a subject would want to limit the impact of her genomic disclosure on her family members, or be asked to have a discussion with specific family members before proceeding. Providing subjects with different levels of genomic anonymity based on their sequence data, along with an estimate of the probability of re-identification and familial impact for each of those anonymity levels, will allow patients to trade off altruistically motivated sharing[21] with privacy consideration, especially when they volunteer to share all the variants in their genome[22].

While the inference accuracy rates are very high, particularly for inferences where Sib_1 _has a homozygous major genotype, we would like to caution that some of these findings are not always highly informative. For example, if the MAF is 0.01, where 99% of the alleles in the population are the major allele, the prior probability for a homozygous major allele is 0.99*0.99 ≅ 0.98. If Sib_1 _has a homozygous major allele, the posterior probability of observing a homozygous major allele in another sibling is (1/4 + 1/4*0.99*0.99 + 1/2*0.99) ≅ 0.99. In this case, the difference between prior and posterior probabilities is only 0.01, and knowledge of the Sib_1 _genotype provides very little information, as most accuracy comes from the allele frequency in the population.

However, homozygous minor alleles are much more informative. With a MAF of 0.2, if Sib_1 _has a homozygous minor genotype, the probability of Sib_2 _having the same genotype, given only the reference population is 0.04. Given that Sib_1 _has a homozygous minor genotype, Sib_2 _will have a homozygous minor allele with probability of (1/4 + 1/4*0.2*0.2 + 1/2*0.2) = 0.36, which is quite different from the prior probability of 0.04.

One limitation of this study is that the population-based estimates for MAF rely on the HapMap study population sizes, which, at present, are small, though these types of sources will continue to expand. For example, the CEPH population contains 90 participants, so each trio child contributes 1/90^th ^of the allele frequency data used in the study. This approach also depends on the independence of the loci considered, and would need to be adapted for SNPs that are in linkage disequilibrium. Extending this study to include linked SNP loci is possible, using the haplotype block information for HapMap populations that is available. To ensure that SNPs are independent, linkage data from the HapMap population can be used to confirm independence, and SNPs that are far from one another may be selected. Additionally, this approach does not consider the possibility of genotypic errors, which may be common on some platforms. An adjustment using a binomial probability distribution could be used to account for possible errors.

## Conclusion

Technologies for sequencing large numbers of SNPs are rapidly dropping in cost, which will help realize the promise of personalized medicine, but pose substantial personal and familial privacy risks. While electronic storage and transmission of genetic tests is not yet a common component of medical record data, these tests will soon be stored in electronic medical records and personally controlled health records[23]. This mandates the need for improved informed consent models and access control mechanisms for genomic data. The increasingly common practice of electronically publishing research-related SNP data requires a delicate balance between the enormous potential benefits of shared genomic data through NCBI and other resources, and the privacy rights of both sequenced individuals and their family members.

## Competing interests

The authors declare there are no competing interests.

## Authors' contributions

CC conceived of the study design, carried out the statistical analysis, generated the figures, and drafted the manuscript. BS carried out experiments using HapMap data and imputed family data. KM helped draft and revise the manuscript, and helped perform the statistical analysis. ZK assisted in conception of the study and critical review of the manuscript. All authors read and approved the final manuscript.

## Appendix

### HapMap CEPH and global population SNP genotypes and allele frequency data

The demographic data used in this project are population-specific SNP allele frequencies from the CEPH HapMap population, Utah residents with ancestry from northern and western Europe, and the global SNP allele frequencies (from all populations that participated in the HapMap)[10] The HapMap project has compiled allele frequency values for a large selection of SNPs – loci in the genome that account for a great deal of genetic variability in populations. Within the CEPH population, there are 30 familial trios, each containing one mother, father, and child. Additionally, the individual genotypes of the 90 CEPH trio participants are directly used in this study. One limitation of this population specific allele frequency database is the small size of each HapMap population – the CEPH population contains 90 participants, and as such, each trio child contributes 1/90^th ^of the allele frequency data that are used in the study.

### Inferring sibling genotypic sequences from HapMap trio children

Here, we explore a specific example of sibling genotypic inference in greater depth, considering the case where one sibling's genotype is known to be *'AA'*, and the goal is to determine the probability that the second sibling's genotype will also be *'AA' *at that locus. The conditional probability expression that sums over the nine possible parental genotypic combinations (for example, maternal genotype *'Aa' *with paternal genotype *'AA'*) at a single SNP, with each specific parental genotypic combination denoted as *i *can be used:

$$\begin{array}{l} p(Sib_{2}AA|Sib_{1}AA)=\sum_{i=1}^{9}p(Sib_{2}AA|parental\;comb.\;i)p(parental\;comb.\;i|Sib_{1}AA)\\ =\sum_{i=1}^{9}\frac{p(Sib_{2}AA\cap parental\;comb.\;i)}{p(parental\;comb.\;i)}p(parental\;comb.\;i|Sib_{1}AA) \end{array}$$

where *Sib*_1_*AA *and *Sib*_2_*AA *refer to Sib_1 _and Sib_2 _genotypes *'AA' *at a selected SNP, respectively.

With unknown parental genotypes, we would calculate *p(Sib*_2_*AA) *considering all nine possible parental genotype combinations, but knowledge that Sib_1 _has genotype *'AA' *allows exclusion of any parental combinations where either parent has genotype *'aa'*, as that would require the transmission of at least one copy of the *'a' *allele to Sib_1_, if non-paternity and new mutations are excluded.

For example, when the child is homozygous major, all possible parental genotypic candidates that involve one or both parent genotypes of *'aa' *are excluded, as it is not possible to have a child with genotype *'AA' *if either parent does not have at least one copy of the *'A' *allele. In this case, there are four possible parental genotypic combinations:

$$\begin{array}{l} =\sum_{i=1}^{4}\frac{p(Sib_{2}AA\cap parental\;comb.\;i)}{p(parental\;comb.\;i)}p(parental\;comb.\;i|Sib_{1}AA)\\ =\left(\frac{p(Sib_{2}AA\cap AA_{M}AA_{F})}{p(AA_{M}AA_{F})}\right)p(AA_{M}AA_{F}|Sib_{1}AA)+\left(\frac{p(Sib_{2}AA\cap AA_{M}Aa_{F})}{p(AA_{M}Aa_{F})}\right)p(AA_{M}Aa_{F}|Sib_{1}AA)\\ +\left(\frac{p(Sib_{2}AA\cap Aa_{M}AA_{F})}{p(Aa_{M}AA_{F})}\right)p(Aa_{M}AA_{F}|Sib_{1}AA)+\left(\frac{p(Sib_{2}AA\cap Aa_{M}Aa_{F})}{p(Aa_{M}Aa_{F})}\right)p(Aa_{M}Aa_{F}|Sib_{1}AA)\\ =(1)(p^{2})+\left(\frac{1}{2}\right)(pq)+\left(\frac{1}{2}\right)(pq)+\left(\frac{1}{4}\right)(q^{2})\\ =p^{2}+pq+\frac{q^{2}}{4}\\ =p^{2}\left[+pq+\frac{q^{2}}{4}\right] \end{array}$$

which allows calculation directly from the SNP population frequencies. Before knowledge of the Sib_1 _genotype was used, *p(Sib*_2_*AA) *would have been the Hardy-Weinberg frequency for major homozygotes, *p*^2^. However, with the Sib_1 _genotype, *p(Sib*_2_*AA|Sib*_1_*AA)*, the additional constraint increases the probability to *p*^2^*+pq+(q*^2^*/4)*, increasing inference accuracy by *pq+(q*^2^*/4)*.

The remaining entries in the probability vector, *p(Sib*_2_*Aa|Sib*_1_*AA)*, and *p(Sib*_2_*aa|Sib*_1_*AA)*, can then be calculated just as we have done for *p(Sib*_2_*AA|Sib*_1_*AA) *above. Again, these probabilities have been generated without any actual knowledge of the parent genotypes. If the Sib_1 _genotype were instead *'Aa' *or *'aa'*, the above technique can similarly be used (with a different combination of possible parental genotypes) to calculate the two other probability vectors, [*p(Sib*_2_*AA|Sib*_1_*Aa)*, *p(Sib*_2_*Aa|Sib*_1_*Aa)*, *p(Sib*_2_*aa|Sib*_1_*Aa)*] and [*p(Sib*_2_*AA|Sib*_1_*aa)*, *p(Sib*_2_*Aa|Sib*_1_*aa)*, *p(Sib*_2_*aa|Sib*_1_*aa)*].

### Validating the sibling genotype probability vector using parental genotypic data

To validate the results of the refining strategy on inferring the second sibling genotype, the authentic parental genotypes are used to create the probability vector *p('AA')*, *p('Aa')*, *p('aa') *at the SNP being evaluated, for the children the pair would be expected to have. For each of the trio pairs at each of the SNPs being tested, the probability vector was calculated.

### Error reduction calculation

The error reduction measurement identifies the extent to which inference error is reduced. For example, where we are trying to infer the probability that Sib_2 _has genotype *'AA' *at a specific SNP, we calculate the absolute value of the difference between our best inference and the Hardy Weinberg probability for Sib_2 _to have genotype *'AA'*, using population-specific allele frequency data and the Sib_1 _genotype, |*p(Sib*_2_*AA|Sib*_1_*genotype)*-*p(Sib*_2_*AA)|*. This value is specifically the improvement to the probability value from the new data, when inferring the specific event that Sib_2 _will have genotype *'AA' *and Sib_1 _will have the specific genotype in question.

Any change to *p(Sib*_2_*AA) *must also correspond with the opposite change in the sum of *p(Sib*_2_*Aa) *and *p(Sib*_2_*aa)*. To accurately represent the overall error reduction by Sib_1 _genotype, with any of three possible Sib_2 _genotypes, the average of the three values is measured. For example, where the Sib_1 _genotype is *'AA'*, the overall average improvement (and error reduction) is the average of |*p(Sib*_2_*AA) - p(Sib*_2_*AA|Sib*_1_*AA)*|, |*p(Sib*_2_*Aa) - p(Sib*_2_*Aa|Sib*_1_*AA)*|, and |*p(Sib*_2_*aa) - p(Sib*_2_*aa|Sib*_1_*AA)*|.

### Scoring metric for calculating correct fraction of inferences

To ascertain whether the inferences are helpful for producing correct answers, a scoring metric was used to calculate the fraction of correct SNP inferences, in our empirical inference validation study. For each SNP inference, the scoring metric provides a full point when the plural entry in the inference vector, (the maximum of *p('AA')*, *p('Aa')*, and *p('aa')*, and thus the predicted sib genotype), matches the plural entry in the parental validation vector (the empirical most likely genotype). Given the parental genotype values, it is possible, and not infrequent, that a validation probability vector has two matching plural values, for example, if *p('AA') *= *p('Aa') *= 0.5. When this is the case, one half point was awarded if the plural value in the inference vector matched one of the two validation choices, to signify that one of the two equally likely candidates was chosen.

There are some conditions that arise from use of a simple scoring metric, where it becomes difficult to score well. For example, a heterozygous Sib_1 _will likely result in a 0.5 score for inferences. A score of 1 point would be possible if one parent had a genotype of *'AA' *and the other had genotype *'aa'*, making the probability that the parents would have a child with genotype *'Aa' *equal 1. Most remaining parental combinations would not result in the probability of child genotype *'Aa' *equal to 1, and would likely result in only a half point. These values can be adjusted using machine learning techniques or more robust decision making algorithms, but those are out of the scope of this work.

## Pre-publication history

The pre-publication history for this paper can be accessed here:


